# The Louse: Its Habits and Methods

**Published:** 1918-06-29

**Authors:** 


### HOW TRENCH FEVER IS CONVEYED.

### The Louse: Its Habits and Methods.

ALL human parasites are repellent and excite feelings of aversion and disgust, and of them all the louse is the most repellent and disgusting. That curious trait in human character that we have several times called attention to, which deters us from even mentioning the name of that which is horrifying or disgusting, is especially prominent in the case of the louse. We refrain from saying that a man is mad, and soothe our feelings by saying that he suffers from nervous breakdown. We shrink from speaking of a water-closet, surely a very inoffensive title, and call it a lavatory, which it is not. We call our madhouses by the ludicrous name of mental hospitals. We speak of bugs as Norfolk Howards; but we have no other name for the louse. There was a time when the louse was so common in all ranks of society that it could not be ignored. It had to be spoken of, but lest the utterance of its name should summon it upon the scene, the name was mispronounced, so that its possessor should not recognise it. Thus those who were infested with the parasite were called, not lousy, but lucy. That this custom of so miscalling lousiness actually prevailed we have the testimony of no less a person than Shakespeare, to whom is attributed the ribald couplet upon the Lucys, on whose deer the poet is said to have made depredations in his youth—

> If lousy is lucy as some folks miscall it,
> Then Lucy is lousy whatever befall it.

A couplet more vigorous in its invective than convincing in its logic.

The louse is not itself uncleanly. Its surface is shining and spotless. It certainly affects uncleanly people, and obviously it is the people rather than the parasite whose names should be expelled from our vocabulary; but such is the injustice of man that we visit upon the cleanly animal the sin of its uncleanly host. However, all these unpleasant things whose names we eschew and ostracise and refuse to mention refuse to be exorcised by such a very simple-minded, not to say stupid, device. And a louse is still a louse, though we refuse to speak of it by any name; and, moreover, as if in revenge for being so long ignored, it has at length compelled us to mention it pretty frequently and to take it into very serious consideration. The louse has long been more than suspected of conveying typhus and relapsing fever, and is now positively proved to convey trench-fever, which is a relapsing fever, and may very probably be the same fever that has for so long received the name of relapsing fever.

### METHOD OF PROPAGATION.

The manner in which the louse conveys trench fever is unique, and one cannot but admire the ingenuity of the microbes, which have resorted to a method of propagation as cleverly adapted to the facts of the situation as any of the elaborate and ingeniously devised stratagems of the orchids described by Darwin. Every other pathogenic microbe that affects humanity gains access to the human body either by being breathed in the air or by being swallowed in the food, or by being inoculated by the bite of some insect, or by settling on some raw surface accidentally produced. It is to the last class that the microbe of trench fever belongs. It is conveyed by the louse, but not, as most insect-borne diseases are, by the bite of the insect. In order to produce its like, and incidentally to produce trench fever, the microbe must gain access to the human body, and must gain access through a wound. In this respect it resembles the microbe of tetanus and others of that class; but the microbe of trench fever lays his plans with far more elaboration and ingenuity than the microbe of tetanus. The latter merely lies in ambush in the superficial layers of the soil, and there waits on the off-chance that some man or beast may fall and graze his skin, or may be wounded by some missile which has previously touched the earth, and so has gathered up a few tetanus microbes for conveyance into the human body. It is evident that this is a very crude and inefficient mode of propagation, and terribly wasteful of microbe life.

### A PITIFUL OUTLOOK FOR MICROBES.

It is pitiful to think of the millions of gnats and midges that swarm in parts of the earth uninhabited by man, and there wait in their millions, generation after generation, for a chance of biting some human being, a chance that never comes or that comes but once in many years, and then only to the insignificant few. But what is this waste of insect life in comparison with the waste of life of such microbes as that of tetanus? The soil of many countries literally swarms with them. There they lie, generation after generation and year after year, waiting, hoping, longing for an opportunity to infect the wound of a human being, an opportunity that to the overwhelming majority of them never comes. They are a stupid race, and their stupid method deserves no better success. Contrast their stupidity with the ingenuity of the trench fever microbe. This artful creature evidently says to itself, "I have to gain access to the human body through some abrasion of the skin. As long as the skin is intact I have no chance of entry. To wait on the off-chance of an accidental abrasion occurring and of my being on the spot to take advantage of it is too tedious and too uncertain. I leave such crude methods to my dull cousins, the microbes of tetanus. My business is to be upon the spot when an abrasion in the skin is made, and therefore I will infest the body of a louse, which produces an intolerable itching, and compels the louse's host to scratch his skin until it is abraded, and so opens a door by which I may enter. In the act of scratching he is sure to crush a louse now and then, and to rub the contents of the louse's

**How Trench Fever is Conveyed**—(*continued*).

body, including myself and my kin, into the abrasion that he has kindly provided for us. In this way we shall leave nothing to chance, but shall compel the clumsy human being to do our work for us and to inoculate himself by his own exertions."

This was the reasoning of the microbe of trench fever, and when put to the test of experience the device proved completely successful. It has this additional advantage—that it is so tortuous and so improbable *primâ facie* that it was very unlikely to be discovered. However, the microbe reckoned without taking into consideration the penetration and skill of Sir David Bruce and his committee, and without regard to the very fine self-sacrifice of eight British soldiers, who offered themselves for experiment, and five of whom contracted trench fever in consequence. Two of these men, named respectively Cole and Edgeler, submitted to be bitten by lice that had previously infected trench fever patients; but Cole and Edgeler contracted no trench fever. They submitted to be bitten every day for a month by 500 lice—think of it!—and yet no trench fever ensued. One of them submitted to be bitten daily for two months, and still no trench fever resulted. Then the experiments were altered. Two more heroes, Seymour and Sullivan, volunteered as victims. Their skins were scarified, and into the scarified area were rubbed the crushed bodies and excreta of lice taken from patients suffering from trench fever. Seymour and Sullivan contracted trench fever, the one after eight days' the other after ten days' incubation. Three other heroes came forward and submitted themselves to martyrdom—Reynolds, Tullett, and Glover. All three had the dried excreta of lice from trench fever cases rubbed into their scarified skins, and all three duly exhibited the typical symptoms of trench fever after seven, eight, and nine days respectively. One more hero, named Ward, submitted to be inoculated with blood from Reynolds, and Ward also exhibited, in five days, the symptoms of trench fever.

The evidence is now complete, and the conclusion that trench fever is conveyed by the inoculation of the excreta of lice cannot be doubted, or even denied, except by anti-vivisectors and such irrational persons. No doubt if the subjects of the experiments had been rats or guinea-pigs, the anti-vivisectors would strenuously deny the validity of the conclusion; but anti-vivisectors have never displayed any tenderness for the vivisection of human beings. On the contrary, their chief delight is in carrying out experiments in moral vivisection on the investigators of disease, of whose investigations the anti-vivisectors are glad to avail themselves when need arises.

